# Anterior cruciate ligament injury: Identifying information sources and risk factor awareness among the general population

**DOI:** 10.1371/journal.pone.0190397

**Published:** 2018-01-05

**Authors:** Yasuharu Nagano, Hiroko Yako-Suketomo, Hiroaki Natsui

**Affiliations:** Department of Sports Wellness Sciences, Japan Women’s College of Physical Education, Tokyo, Japan; University Hospital Modena and Reggio Emilia, ITALY

## Abstract

**Introduction:**

Raising awareness on a disorder is important for its prevention and for promoting public health. However, for sports injuries like the anterior cruciate ligament (ACL) injury no studies have investigated the awareness on risk factors for injury and possible preventative measures in the general population. The sources of information among the population are also unclear. The purpose of the present study was to identify these aspects of public awareness about the ACL injury.

**Materials and methods:**

A questionnaire was randomly distributed among the general population registered with a web based questionnaire supplier, to recruit 900 participants who were aware about the ACL injury. The questionnaire consisted of two parts: Question 1 asked them about their sources of information regarding the ACL injury; Question 2 asked them about the risk factors for ACL injury. Multivariate logistic regression was used to determine the information sources that provide a good understanding of the risk factors.

**Results and discussion:**

The leading source of information for ACL injury was television (57.0%). However, the results of logistic regression analysis revealed that television was not an effective medium to create awareness about the risk factors, among the general population. Instead “Lecture by a coach”, “Classroom session on Health”, and “Newspaper” were significantly more effective in creating a good awareness of the risk factors (p < 0.001).

## Introduction

Anterior cruciate ligament injury is a critical sports injury, with far reaching consequences. In the United States, there are around 200,000 cruciate ligament injuries annually [1]. Among Japanese junior high and high school athletes, about 3,000 ACL injuries occur annually, and the injury rate is 0.80 per 1000 athletes [2]. Following the injury, most cases require a reconstruction of the ACL and long-term rehabilitation. Most articles published in recent years, advocate a return to unrestricted sports 6 months or later following ACL reconstruction [3]. Moreover, long-term studies have reported that about 50% of patients develop osteoarthritis of the knee joint, 15 years after an ACL injury, irrespective of the treatment [3,4,5]. Almost all people who participate in any kind of sports activity are at a risk for ACL injury. Therefore, this injury carries a pressing concern in sports medicine, and needs effective prevention strategies.

Previously, many researchers have investigated the mechanisms, risk factors, and prevention methods for ACL injury. Based on the outcomes of these studies, a consensus about ACL injury prevention has recently been reported [3]. Through research, the mechanisms, risk factors, and prevention methods for ACL injury were gradually understood. Almost 80% of the ACL injuries are non-contact in nature. Injuries often occur when landing from a jump, cutting or decelerating [3]. Well-designed injury prevention programs, which focus on proper landing and side-step cutting movement techniques reduce the risk of ACL in athletes, particularly women [3]. However, the incidence of ACL injury did not change between 2005 and 2013 in Japan [2]. One probable reason could be that the existing knowledge did not percolate to the general population, and awareness about the risk factors and prevention methods was insufficient. Thus, disseminating this information is necessary.

For public health, increasing the awareness about a disorder is important for prevention. There have been surveys on public awareness or beliefs about cancer in several countries [6,7,8]. Increasing the awareness through public health policies enables prevention by screening. However, for a sports injury like the ACL injury, no study has investigated the public awareness about its risk factors and prevention methods, to the best of our knowledge. The most effective sources of information for creating awareness are also unclear. If these can be identified, they can be used to spread awareness and adequate understanding.

Therefore, the purpose of this study was to understand how the general population acquires information about ACL injury and to evaluate the most effective medium for understanding the risk factors associated with it. It was hypothesized that the general population acquired information about ACL injury from mass media (television and internal sources); however, these information sources did not necessarily provide a good understanding of the risk factors.

## Materials and methods

### Study setting

The present study was a cross-sectional analysis of the data obtained from a web-based questionnaire survey. The questionnaire was randomly distributed among the people registered with a web questionnaire supplier (Rakuten Research Inc.), to recruit 900 participants who were aware about ACL injury. According to the power analysis [9], the required sample size for a descriptive study of the dichotomy variable (P = 0.50, W = 0.10, confidence level = 95%) was 384, and required sample size for difference of ratio of dichotomy variable (smaller P_1_ = 0.45, difference of P = 0.10, α = 0.05, β = 0.20) was 407. Based on these values, we targeted a sample size of 900. Participants were assigned equitably according to sex (men and women) and age (20’s, 30’s, 40’s, and 50’s). While rolling out the survey, a web questionnaire supplier invited 310,325 affiliates to participate in the survey. When the number of respondents who participated and were aware of the ACL injury reached 112 or 113 for each gender in all age groups, and 900 in total, the survey was closed. The survey achieved the desired sample size in 2 days (June 20–21, 2016). Demographic characteristics of the study populations are shown in Table 1. Participants who agreed to participate in this survey answered the questionnaire voluntarily, and information was collected anonymously, without revealing the identity of any individual participant. The ethical review board of Japan women’s college of physical education approved the present study, and the study was conducted based on the principles in the Declaration of Helsinki.

**Table 1:** Demographic characteristics of the study populations.

	Number	Age							
		Mean	Median	SD	[range]				
Total	900	40.2	40.0	10.9	[	20	―	59	]
Males	450	40.5	40.0	11.0	[	20	―	59	]
20―29y	112	25.9	27.0	2.6	[	20	―	29	]
30―39y	112	36.0	36.0	2.5	[	30	―	39	]
40―49y	113	45.3	46.0	2.8	[	40	―	49	]
50―59y	113	54.5	55.0	2.7	[	50	―	59	]
Females	450	40.0	40.0	10.8	[	20	―	59	]
20―29y	112	26.1	27.0	2.7	[	20	―	29	]
30―39y	112	35.2	36.0	3.1	[	30	―	39	]
40―49y	113	44.4	44.0	2.7	[	40	―	49	]
50―59y	113	54.1	54.0	2.7	[	50	―	59	]

### Questionnaire

The questionnaire consisted of one screening question and two main questions (S1 File). The screening question assessed whether the respondent was aware about the ACL injury. If the answer was positive, the questionnaire continued to question 1 and 2. Those who answered in the negative for the screening question were dropped from the survey. Question 1 asked the respondent about the source of information about ACL injury. Participants selected their answers from a list of given options. The options were, “Injury to self”, “Injury among family or relatives”, “Injury among friends”, “Lecture from family”, “Lecture from coach”, “Classroom session on Health”, “Any Classroom session, except on health”, “Television”, “Magazine”, “Comics”, “Internet”, “Newspaper”, and “Poster or flyer in the hospital”. Participants were allowed to select multiple answers. Question 2 assessed their knowledge about risk factors for ACL injury. Participants answered “likely to be a risk for ACL injury” or “not likely to be a risk for ACL injury” for each factor. Factors were “Bone geometry”, “ACL size”, “Joint laxity”, “Hormone”, “Flexibility”, “Foot pronation”, “Weakness of front thigh (quadriceps)”, “Weakness of back thigh (hamstrings)”, “Weakness of hip muscles”, “Poor single limb balance”, “Increase of weight”, “Drinking”, “Smoking”, “Genu valgum during landing”, and “Genu varum during landing”. Participants were allowed to select multiple answers. We considered nine factors as risk factors, based on previous studies, and scored the answers for a maximum of nine points. Proven risk factors were “Bone geometry” [10], “ACL size” [10], “Joint laxity” [10], “Hormone” [10], “Foot pronation” [10], “Weakness of back thigh (hamstrings)” [3], “Weakness of hip muscles” [3,11], “Poor single limb balance” [12], and “Genu valgum during landing” [3,10]. We evaluated the validity of this questionnaire and scored points in the colloquium, which consisted of sports medicine physicians, physical therapists and public health personnel. If the sum of the average score and 1SD exceeded nine points, it was considered as a ceiling effect.

### Statistical analysis

We allocated participants to two groups: the high understanding group (over 7 points) and low understanding group (under 4 points), based on the score of question 2. Participants scoring 5 or 6 points were excluded from the analysis to accentuate the difference between groups. To determine the source of information, which provides a good understanding of the risk factors, we examined the association between the two groups and each source of information, using chi-square test or Fisher exact test. Moreover, multivariate logistic regression was performed to determine the sources of information that provide a good understanding of the risk factors. Input variables were selected from the significant factors based on the results of the chi-square test or Fisher exact test (p<0.1). Multivariate logistic regression was conducted using forward selection (likelihood ratio), and model chi-square test (p<0.05). Overall percentage of correct information provided, goodness-of-fit (p>0.05), and odds ratio of each factor in the final regression model was calculated. All statistical analyses were performed using SPSS Statistics Version 19.0 for Windows (IBM; Brush Prairie, WA, USA).

## Results

### Attributes of participants

Total 4248 people responded to the screening question and 900 participants who were aware of the ACL injury completed the survey.

### Source of information about ACL injury

Sources of information about ACL injury are shown in Table 2. The most frequent source was “Television” (57.0%), followed by “Injury to friends” (22.4%), “Lecture from coach” (16.6%), and “Internet” (16.3%).

**Table 2:** Information source of ACL injury.

Source	Response (n = 900)	Percentage (%) [95%CI]					
Injury to self	62	6.9	[	5.2	―	8.5	]
Injury to family or relative	65	7.2	[	5.5	―	8.9	]
Injury to friends	202	22.4	[	19.7	―	25.2	]
Lecture from family	25	2.8	[	1.7	―	3.9	]
Lecture from coach	149	16.6	[	14.1	―	19.0	]
Classroom session on health	34	3.8	[	2.5	―	5.0	]
Any classroom session, except on health	21	2.3	[	1.3	―	3.3	]
Television	513	57.0	[	53.8	―	60.2	]
Magazine	40	4.4	[	3.1	―	5.8	]
Comics	23	2.6	[	1.5	―	3.6	]
Internet	147	16.3	[	13.9	―	18.7	]
Newspaper	84	9.3	[	7.4	―	11.2	]
Poster or flyer in the hospital	34	3.8	[	2.5	―	5.0	]

### Understanding of the risk factors for ACL injury

**Fig 1:**
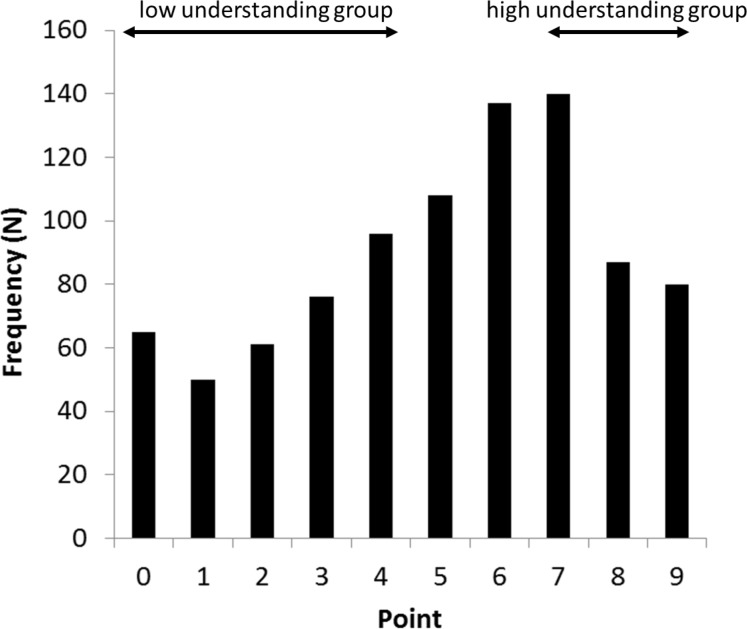
Distribution of scores for risk factors of ACL injury.

The understanding of the risk factors for ACL injury is shown in Table 3. The understanding of “Hormone” and “Foot pronation” was low (20.4% and 28.2%, respectively). The distribution of the score for risk factors is demonstrated in Fig 1. The average score for the risk factors proven by previous studies was 5.1 (median = 5, s = 2.61) out of a maximum of nine points possible. There was no ceiling effect in this questionnaire. In addition, Cronbach α was 0.80.

**Table 3:** Responses about risk factors for ACL injury.

	Response (n = 900)	Percentage (%) [95%CI]					
Bone geometry ※	462	51.3	[	48.1	―	54.6	]
ACL size ※	602	66.9	[	63.8	―	70.0	]
Joint laxity ※	613	68.1	[	65.1	―	71.2	]
Hormone ※	184	20.4	[	17.8	―	23.1	]
Flexibility	717	79.7	[	77.0	―	82.3	]
Foot pronation ※	254	28.2	[	25.3	―	31.2	]
Weakness of front thigh (quadriceps)	623	69.2	[	66.2	―	72.2	]
Weakness of back thigh (hamstrings) ※	620	68.9	[	65.9	―	71.9	]
Weakness of hip muscles ※	600	66.7	[	63.6	―	69.7	]
Poor single limb balance ※	637	70.8	[	67.8	―	73.7	]
Increase of weight	723	80.3	[	77.7	―	82.9	]
Drinking	195	21.7	[	19.0	―	24.4	]
Smoking	171	19.0	[	16.4	―	21.6	]
Genu valgum during landing ※	570	63.3	[	60.2	―	66.5	]
Genu varum during landing	566	62.9	[	59.7	―	66.0	]

※The cell done a highlight of by gray was treated as a risk factor.

### Information sources influencing the understanding of risk factors

From the score of question 2, we extracted the high understanding group (n = 348) and the low understanding group (n = 307). Based on the results of the chi-square test or Fisher exact test, “Injury to family or relative”, “Lecture from the coach”, “Classroom session on Health”, “Magazine”, “Newspaper”, and “Poster or flyer in the hospital” achieved a good score (above 7 points) on the recognition of risk factors. The results of multivariate logistic regression analysis of information sources providing good recognition of risk factors are shown in Table 4. The final regression model included “Lecture from the coach”, “Classroom session on Health,” and “Newspaper”, and provided a significantly good information of risk factors (p<0.001) and had goodness-of fit (= 0.565).

**Table 4:** Results of multivariate logistic regression analysis about the sources of information for awareness of risk factors. (N = 639).

	Coefficients	P value	Odd Ratio [95% CI]					
Lecture from coach	0.671	0.003	1.957	[	1.258	―	3.042	]
Class of Health	1.034	0.036	2.813	[	1.069	―	7.403	]
Newspaper	0.556	0.052	1.743	[	0.996	―	3.052	]
Constant	-0.369	0.000	0.691					

Model chi-square test p<0.001

Overall percentage of correctly predicted: 58.4%

Goodness-of fit (Hosmer and Lemeshow test) = 0.565

## Discussion

The present study investigated the general awareness about ACL injury in Japan using a web-based questionnaire. The main findings of this study were that the most important source of information was television, but this medium was not effective in building awareness in the general population. It was found that people only hear about "ACL injury" from the news or documentaries of television; the awareness of risk factors and prevention is not included in the broadcast. To increase the understanding about ACL injury, dissemination of risk factors and prevention methods through the television is necessary. If the scientific societies or sports organizations can provide correct information through the television, awareness and understanding about ACL injury could be created in the general population.

Other information sources that contributed to the understanding of risk factors were lectures from a coach, classroom session on health, and newspaper. These sources were found to be effective for understanding the injury; however, they were less frequently used. These sources should be utilized to disseminate information. With regard to awareness among coaches, Norcross et al. [13] investigated their knowledge on injury prevention programs and found that 52% reported being aware of injury prevention programs. Interestingly, the most important source of information on injury prevention programs among players was their coaches. Indeed, players were 4.94-times more likely to be aware of prevention programs if their coaches were aware of the programs [14]. To improve the dissemination of injury prevention, all coaches should understand the risk factors and convey this information to the players. In the Japanese junior high school class of health, a chapter is included on injury prevention [15]; however, it does not address a specific injury. We hope that the class of health covers the ACL injury as a representative sports injury. The newspaper is an effective source to disseminate the knowledge; however, the readership is declining. Today, internet could be an effective substitute for a newspaper. In a dissemination study of rugby injury prevention programs [14], social media was also found to be a significant contributor to knowledge among coaches and players. To spread the understanding of ACL injury, an increase in the opportunity to access these information sources through social media is probably necessary.

The limitation of this study is that it used a web-based questionnaire survey. Therefore, we could not identify the population surveyed and participation bias. There is also a possibility of some participants providing false answers. However, this web-based survey is a useful tool for a wide-reaching public investigation in a short period. In addition, to recruit 900 participants who knew of ACL injury, 4248 people were screened, which indicated that 21.2% had heard about it. Although awareness on sports injury or prevention programs among coaches or players has been investigated in previous studies [13,14], few studies have investigated awareness in the general population. Moreover, even fewer studies have done so using a web-based questionnaire. Therefore, these results cannot be compared with other injuries. In the future, additional survey studies are expected as more evidence becomes available.

In conclusion, the results of the present study demonstrated that the most frequent source of information regarding ACL injuries was television, but it did not contribute to the understanding of risk factors. A lecture from the coach, classroom session of health, and newspapers, contributed to the understanding of risk factors. It is recommended to provide improved information through the television or to increase the opportunity for people to attend a lecture by a coach, a classroom session on health, and access newspapers, to increase the awareness and good understanding of ACL injury in the general population.

## Supporting information

S1 File Questionnaire. (DOC)
