# Supplementary material for: Anterior cruciate ligament injury: Identifying information sources and risk factor awareness among the general population
Source: PLoS One. 2018 Jan 5;13(1):e0190397. doi: 10.1371/journal.pone.0190397 (PMC5755787; doi:10.1371/journal.pone.0190397)
Supplement: S1 File — (DOC) [file pone.0190397.s001.doc]

[Screening question]

膝関節のケガの中で，前十字靱帯という靱帯の損傷（前十字靱帯損傷）を聞いたことがありますか．

(Among knee injuries, are you aware of the Anterior Cruciate Ligament (ACL) injury?)

1．ある　(Yes)

2．ない　(No)

[Question1]

どこで前十字靱帯損傷を耳にしましたか．

How did you learn about the ACL injury?)

1．自分自身がその怪我をしたことがある　(Injury to self)

2．家族や親戚がその怪我をしたことがある　(Injury to family or relatives)

3．友人・知人(家族親戚以外)がその怪我をしたことがある　(Injury to friends)

4．家族の話　(Lecture from family)

5．スポーツ指導者(部活，スポーツクラブ)の話　(Lecture from coach)

6．学校での保健の授業　(Classroom session on Health)

7．学校での保健以外の授業　(Any Classroom session, except on health)

8．テレビ　(Television)

9．雑誌　(Magazine)

10．マンガ本　(Comics)

11．インターネット　(Internet)

12．新聞　(Newspaper)

13．病院のポスターやチラシ　(Poster or flyer in the hospital)

14．その他(　　　　　)　(Others)

[Question2]

下記の項目それぞれについて，前十字靭帯損傷の原因になりそうなものを選択してください．(Please select “likely to be a risk factor for ACL injury” or “not likely to be a risk factor for ACL injury” for each factor.)

・膝の骨の形 (Bone geometry)

1. なりそう　(likely to be a risk factor for ACL injury)

2．なりそうでない　(not likely to be a risk factor for ACL injury)

・靭帯の太さ (ACL size)

1. なりそう　(likely to be a risk factor for ACL injury)

2．なりそうでない　(not (likely to be a risk factor for ACL injury)

・関節のゆるさ (Joint laxity)

1. なりそう　(likely to be a risk factor for ACL injury)

2．なりそうでない　(not (likely to be a risk factor for ACL injury)

・ホルモン (Hormone)

1. なりそう　(likely to be a risk factor for ACL injury)

2．なりそうでない　(not (likely to be a risk factor for ACL injury)

・身体の硬さ，柔軟性 (Flexibility)

1. なりそう　(likely to be a risk factor for ACL injury)

2．なりそうでない　(not (likely to be a risk factor for ACL injury)

・扁平足 (Foot pronation)

1. なりそう　(likely to be a risk factor for ACL injury)

2．なりそうでない　(not (likely to be a risk factor for ACL injury)

・太ももの前側の筋肉(大腿四頭筋)の弱さ (Weakness of front thigh (quadriceps))

1. なりそう　(likely to be a risk factor for ACL injury)

2．なりそうでない　(not (likely to be a risk factor for ACL injury)

・太ももの後ろ側の筋肉(ハムストリングス)の弱さ (Weakness of back thigh (hamstrings))

1. なりそう　(likely to be a risk factor for ACL injury)

2．なりそうでない　(not (likely to be a risk factor for ACL injury)

・股関節の筋肉の弱さ (Weakness of hip muscles)

1. なりそう　(likely to be a risk factor for ACL injury)

2．なりそうでない　(not (likely to be a risk factor for ACL injury)

・片脚バランスの悪さ (Poor single limb balance)

1. なりそう　(likely to be a risk factor for ACL injury)

2．なりそうでない　(not (likely to be a risk factor for ACL injury)

・体重の増加 (Increase of weight)

1. なりそう　(likely to be a risk factor for ACL injury)

2．なりそうでない　(not (likely to be a risk factor for ACL injury)

・飲酒 (Drinking)

1. なりそう　(likely to be a risk factor for ACL injury)

2．なりそうでない　(not (likely to be a risk factor for ACL injury)

・喫煙 (Smoking)

1. なりそう　(likely to be a risk factor for ACL injury)

2．なりそうでない　(not (likely to be a risk factor for ACL injury)

・着地の際のX脚 (Genu valgum during landing)

1. なりそう　(likely to be a risk factor for ACL injury)

2．なりそうでない　(not (likely to be a risk factor for ACL injury)

・着地の際のO脚 (Genu varum during landing)

1. なりそう　(likely to be a risk factor for ACL injury)

2．なりそうでない　(not (likely to be a risk factor for ACL injury)
